# Measurement of Mechanical Properties of Cantilever Shaped Materials

**DOI:** 10.3390/s8053497

**Published:** 2008-05-26

**Authors:** Eric Finot, Ali Passian, Thomas Thundat

**Affiliations:** 1 Institut Carnot de Bourgogne, UMR 5209 CNRS-Université de Bourgogne, 9 Av. A. Savary, BP 47 870, F-21078 Dijon Cedex, France; 2 Nanoscale Science and Devices, Oak Ridge National Laboratory, Oak Ridge, TN 37831, USA; 3 Department of Physics, University of Tennessee, Knoxville, TN 37996, USA; E-mails: passianan@ornl.gov; thundattg@ornl.gov

**Keywords:** Microcantilever, mechanics, ageing, environment, stress, gas, materials, sensor, pressure, temperature

## Abstract

Microcantilevers were first introduced as imaging probes in Atomic Force Microscopy (AFM) due to their extremely high sensitivity in measuring surface forces. The versatility of these probes, however, allows the sensing and measurement of a host of mechanical properties of various materials. Sensor parameters such as resonance frequency, quality factor, amplitude of vibration and bending due to a differential stress can all be simultaneously determined for a cantilever. When measuring the mechanical properties of materials, identifying and discerning the most influential parameters responsible for the observed changes in the cantilever response are important. We will, therefore, discuss the effects of various force fields such as those induced by mass loading, residual stress, internal friction of the material, and other changes in the mechanical properties of the microcantilevers. Methods to measure variations in temperature, pressure, or molecular adsorption of water molecules are also discussed. Often these effects occur simultaneously, increasing the number of parameters that need to be concurrently measured to ensure the reliability of the sensors. We therefore systematically investigate the geometric and environmental effects on cantilever measurements including the chemical nature of the underlying interactions. To address the geometric effects we have considered cantilevers with a rectangular or circular cross section. The chemical nature is addressed by using cantilevers fabricated with metals and/or dielectrics. Selective chemical etching, swelling or changes in Young's modulus of the surface were investigated by means of polymeric and inorganic coatings. Finally to address the effect of the environment in which the cantilever operates, the Knudsen number was determined to characterize the molecule-cantilever collisions. Also bimaterial cantilevers with high thermal sensitivity were used to discern the effect of temperature variations. When appropriate, we use continuum mechanics, which is justified according to the ratio between the cantilever thickness and the grain size of the materials. We will also address other potential applications such as the ageing process of nuclear materials, building materials, and optical fibers, which can be investigated by monitoring their mechanical changes with time. In summary, by virtue of the dynamic response of a miniaturized cantilever shaped material, we present useful measurements of the associated elastic properties.

## 1. Introduction

Microcantilevers were first designed and fabricated for use as force sensors. Possessing an extremely high force sensitivity, in the piconewton (pN) range, the cantilevers have made Atomic Force Microscopy (AFM) [1] universally recognized not only as a versatile microscopy technique with high spatial resolution, but also as a powerful tool for measuring the forces between surfaces. Using conventional micromachining techniques it is possible to fabricate cantilevers with desired spring constants, and therefore, force sensitivity. Availability of inexpensive, mass-produced cantilevers also triggered applications other than imaging, where cantilevers act as physical, chemical, and biological sensors. It has been observed that the bending of a cantilever is influenced by ambient conditions such as relative humidity and temperature. In 1994, researchers reported a novel exploitation of these undesirable effects in imaging applications and laid the basis for the development of highly sensitive sensors for vapor adsorption and measurement of changes in temperature [2, 3]. These early observations later lead to the development of a unique family of mechanical sensors with numerous new applications in physical, chemical and biological sensing. The miniature size and the simple structure of a cantilever, together with its ability to operate in different ambient conditions such as liquids, gases, and vacuum, make the cantilever a versatile sensor platform. Since the cantilevers can also be modified to detect electromagnetic fields and forces, they find applications in many aspects of physical sensing.

A cantilever sensor can be operated in two different modes: the static mode, where the cantilever deflection is monitored, and the dynamic mode, where the cantilever resonance is monitored. The deflection of a cantilever can be due to number of processes such as molecular adsorption, thermal effects, electric and magnetic fields, and fluid flow. Adsorption-induced deflections are attributed to changes in the surface free energy and are observed only when a differential adsorption occurs between the cantilever surfaces. Depending on the mode of operation, several methods for reading the movement of a cantilever have been developed. These readout techniques can be applied to a single cantilever or to arrays of cantilevers.

One of the first applications of the microcantilever was in sensitive mass balance measurements, where it served as a micro resonator. As a result, a mass resolution in the picogram range was achieved [4], which outperformed the thermogravimetric approaches by five orders of magnitude. Cantilevers shorter than 10 μm in length with sub-attogram sensitivity were demonstrated in 2004 [5-7], enabling the detection of single virus particles of femtogram mass [8]. Optimization results in an increase of the Q-factor of the nanoresonators [9], and values as high as 10,000 can be achieved. The use of higher order modes for short cantilevers [10] is also critical. Operation at frequencies as high as 1.5 MHz enables a theoretical mass resolution of approximately 20 ag/Hz [11].

Based upon the simultaneous measurements of bending and resonance frequency, a miniature magnetic force balance was developed by Finot *et al.* to measure the magnetic susceptibility of nanogram quantities of powders [12]. Here the cantilever with nanogram amount of magnetic material acts as a Faraday balance.

Applications of microcantilevers as label free biological and chemical sensors have been demonstrated by many groups. Good overviews of the early work have been written by Raiteri and Thundat [13, 14]. More recent works have been covered by Lavrik [15] and others [6, 16-18]. Briefly, when the molecular adsorption is confined to a single surface of a microcantilever, the molecular interactions can be studied by: (i) noting a shift in the resonance frequency and (ii) monitoring the bending. The latter offers an advantage over other acoustic sensors (QCM, SAW) by providing an additional measurable physical quantity: the surface stress caused by the forces involved in the adsorption process. In molecular recognition experiments using an array of cantilevers, adsorption enhancement is achieved by coating each cantilever sensor with a different sensitive layer allowing the array-device to operate as an artificial chemical nose [19, 20].

The ability of microcantilevers to function as a bioassay was demonstrated by the detection of prostate cancer disease [21, 22]. In another example, the specificity of enzymes was utilized to construct a highly selective glucose biosensor [23, 24]. Immunosensors for bacterial organisms [25], virus [8, 26], *Bacillus anthraces* [27], pesticides [17] or antibodies [28, 29] and proteins [30], as well as DNA sensors [18, 31, 32], have been reported. Similarly, many chemical sensors [33] have been demonstrated, for example for low level caesium detection [34].

Microcantilevers were also found to be particularly suitable for the chemical sensing of vapours and gases [35-37]. Hydrogen, for example, can be detected by its adsorption on a Platinum-coated sensor [2, 38]. Likewise, hydrogen fluoride can be detected using a silica microcantilever both in liquid phase at femtomolar concentrations [2, 39, 40] and in gas phase [40]. Other examples include the detection of various alcohols using polymers such as PMMA or PDMS [41] and quantification of individual components in a gas mixture [42]. Quantitative measurements of the concentration of metal ions in aqueous solutions down to 10-10 M [43] using ion-selective SAM-modified microcantilevers [34], as well as the measurement of pH values of solutions with a sensitivity of around 50 nm deflection per pH unit 10 have also been achieved.

Another application, where the use of microcantilevers has not been fully exploited, is the study of mechanical properties of materials. Microcantilevers have proven to be powerful tools for the investigation of the mechanical properties of microsystems that is otherwise unattainable, or not easily achievable by other more macroscopic approaches [44]. A variety of methods already exist for the measurement of the elastic properties of thin films. Well-established measurement techniques for the properties of macrostructures include ultrasound [45], rheology [46], and tribology [47]. Ultrasonic wave techniques require centimetre-sized samples, which do not ensure the sample homogeneity. Techniques based on the nanoindentation of the sample surface can accurately measure the local surface stiffness, but not the bulk mechanical properties. A review of the existing methods indicates that the conventional techniques are not readily suitable for *in-situ* investigations of micrometer-sized sample volumes and therefore, the determination of these parameters in the field of microtechnology remains quite difficult.

The determination of physical parameters is of significant interest for optimizing the design of mechanical structures. The accurate measurement of mechanical properties is contingent upon a rigorous understanding of the length scale dependence. The natural length scale will depend on such structural features of the material as the average grain size, and the dislocation length. To further classify the material properties, one may distinguish the “thin” microstructure, with length scales below a grain size, from a “thick” microstructure or a macrostructure, with length scales encompassing many grains.

In order to optimize the performance of the meso and macro-scale devices, material engineers have recognized the need for a better understanding of the processes in the micro-domain. Such optimization efforts require similar investigations of the mechanical properties at the nanoscale. Five parameters are usually used to characterize the mechanical response of the material:

The *Young modulus* (E) is the primary measure of the stiffness of the material. It is defined for small *strains* (e) as the rate of change of *stress* (s) with strain, that is, *E* = σ/ ε. Stress [48-50] can be induced thermally in thin film multilayer structures due to the difference in the thermal expansion coefficients between the adjacent layers, or because the structures are subjected to temperature changes during their manufacturing and subsequent use.

The *Poisson ratio* (ν) is the ratio between the transverse strain (contraction normal to the applied load) and the axial strain (extension).

The *yield strength* (σ_Y_) corresponds to the stress at which the material gets plastically deformed. It depends on the rate of deformation (strain rate) and, more significantly on the temperature *T* and the microstructure (grain size).

The *fracture strength* (σ_F_) is the stress leading to the beginning of fracture.

The *residual stress* (σ_R_), which is often neglected [51], but can also lead to the bending and buckling. It is characterized by two quantities: its magnitude (σ_R_) and its gradient ∇(σ_R_).

For thin mechanical structures, the elastic (E, ν) or inelastic response (s) play a major role. The viscoelastic properties of silicon microcantilevers can usually be avoided. However, amorphous solids such as glass, polymers can be characterized with a viscosity η (10^18-21^ Pa.s for glass) in the plastic regime. The viscosity is defined mathematically as the ratio of the shearing stress to the velocity gradient in the material, i.e., the material's resistance to flow.

Continued miniaturization of mechanical structures will lead to increased influence of surface stress [21], and these effects have found applications in, for example, electrochemistry [52, 53], and actuators [54], among others. In resonance frequency measurements, often changes in the surface stress, for instance induced by adsorption [55] or/and a viscous environment [56], can give rise to a noticeable change in the spring constant resulting in a miscalculation of the mass loading. Quantum mechanical calculations show that, in a viscous solution, the frequency shift of the nanoscale cantilever can be determined from the change in the surface stress that is generated by the biomolecular interaction with negligible contributions from the ensuing mass loading by the bio molecules [56]. A general scaling law connecting the stiffness and dissipative properties of a linear mechanical oscillator immersed in a viscous fluid has been derived [57, 58].

While, many mechanical properties of macrostructures may exist in handbooks, as shown in Table 1, there is still no database for thin structures. For instance, Young's modulus of amorphous silicon differs largely from the single-crystal phase (400 GPa).

Unlike other mechanical oscillators, an advantage of the cantilever may be that it is not limited to only one type of material. For example, fabrication of SAW devices is restricted to piezoelectric substrates. Silicon was initially the material of choice in the microfabricated devices because of its favorable electrical and mechanical properties, enabling inexpensive, batch-fabricated, high-performance sensors and transducers that could be easily interfaced with advanced microelectronics [6, 43, 59-62].

Recently polymers have been applied in the fabrication of microdevices because of their desirable properties (e.g. biocompatibility and cost) [63-65]. Cantilevers can be fabricated using the polymer SU-8, thereby providing the sensors with very high sensitivity due to convenient mechanical material properties [66-68]. The fabrication process for polymer cantilevers is based on spin coating of the photosensitive polymers and near-ultraviolet lithography [67], allowing well-controlled and uniform mechanical properties for the cantilevers. The elastic constants of such cantilevers have been measured, and their dynamic response has been studied [69, 70].

Metallic microcantilever beams of various thicknesses and lengths have been fabricated by bulk micromachining [54, 69] using thin films of silver [71], and iron [72], or electrodeposition [26, 73, 74]. As a result, the inelasticity of thin metal films could be studied [75]. Palladium cantilevers were used mostly for hydrogen detection [76, 77].

Other materials used in cantilever and micromechanical oscillator fabrication include, glass [78] or amorphous carbon (ta-C) [79], suitable for the study of mechanical dissipation mechanisms in such materials [80]. Exotic materials such as cement [81], wood [82] and rubber [83] have also been investigated using cantilevers.

Microfabrication techniques for cantilevers [85, 86] can be categorized into surface or bulk micromachining, which are based on IC manufacturing technologies. Surface micromachining is an additive process, which consists of fabricating micromechanical structures from deposited thin films, such as silicon nitride, polycrystalline silicon and other materials [78]. Bulk micromachining, such as laser lithography [87] or focused ion beam [23] is a subtractive process that uses the selective removal of significant amounts of silicon, or other materials, from a substrate to form microstructures.

Here we review our recent research on the measurement of the mechanical properties of cantilever shaped materials. First, we present the advantage of the small size of the cantilever real-time, in-situ measurement of their mechanical properties. Then, we will introduce the necessary theoretical background of the elastic and inelastic parameters for discerning the bulk and surface mechanical properties. We also discuss the limitations of the continuum mechanics as well as the effect of the environment such as the pressure and the temperature. Finally, we will discuss applications in various areas such as the ageing process in nuclear plant, the setting of cement or the etching or swelling of coatings.

## 2. *In situ* measurements

One of the underutilized capabilities of a microcantilever is its ability to carry out *in-situ* measurements of mechanical properties. Here we will begin with a description of the experimental setup used for carrying out such measurements. Due to its micrometric size, the cantilever can easily be inserted into or integrated with a small vessel or cell.

### Static Measurements

**Figure 1.**
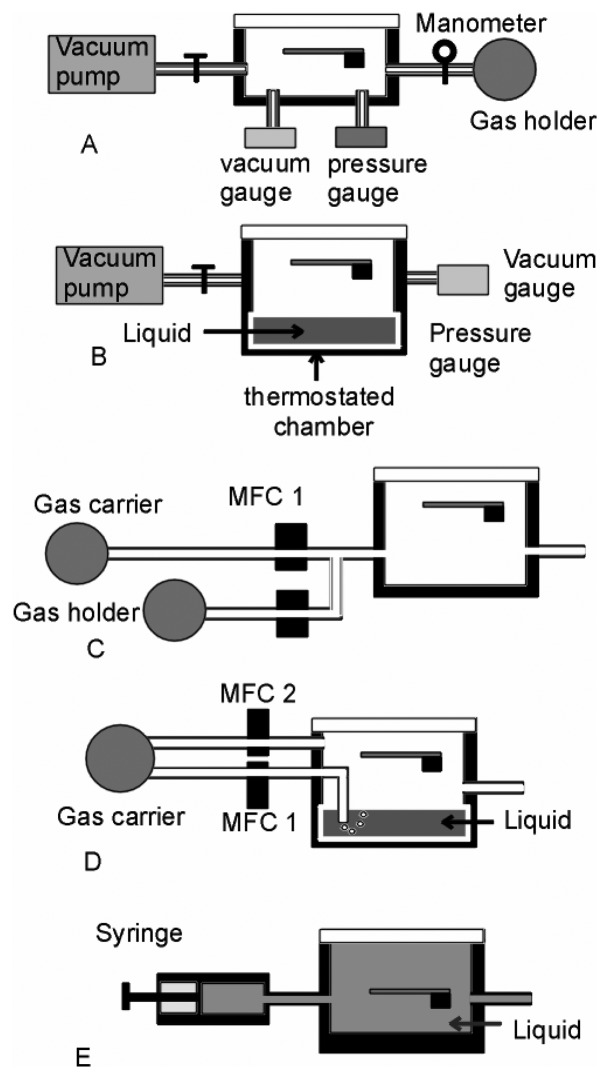
Schematics of the setup for in-situ measurements of the mechanical properties in gaseous environment in static (A-B), under flow (C-D) and in liquid (E).

For static gas measurements, a prior vacuum is often required. A special cell holding the cantilever can be designed easily in the laboratory to withstand pressures ranging from a secondary vacuum to almost 15 bar [2, 88]. The cell made of aluminium alloy (Figure 1, A-B) has a low mass of about 1 g and a small internal volume of 0.5 cm^3^. The pumping, achieved using a primary pump in conjunction with a turbo molecular pump, permits achieving pressures down to 10^-8^ bar at the input of the capillary tube. Heating of the cantilever is achieved electrically using wires wrapped around the cell with a proper temperature calibration. Such a setup permits the isolation of the cell from the pumping system. Vapors can be introduced into the system using a liquid tube connected to the vacuum cell. The vapor, emanating from a small amount of liquid kept at room temperature in a tube, expands throughout the enclosed volume of the cantilever cell.

### Dynamic Measurements

For dynamic gas measurements, a control of the gas flow is required (Figure 1, C-D), C-D) [40]. The gas molecules to be detected are first concentrated in a tank compressed at a pressure of around 100 bars and then diluted using a carrier gas. Automated mass flow controllers (MFC) coupled with solenoid valves allow monitoring and mixing the gas flow. The ratio between the gas flow rate *V*_gas_ at the tank output and the total flow rate *V*=*V*_gas_ + *V*_carrier_ defines the gas concentration *C* at the inlet of the measurement cell. In dynamics, the time *t_dyn_* to complete the adsorption process can be regulated using the flow rate *V*:

(1) $$t_{\text{dyn}}=\frac{W}{C}\left(\frac{S}{V}\right)$$

where, *W* could be viewed as the adsorption capacity of the cantilever (molecules/μm^2^), *C* is the vapor inlet concentration (molecules/liters), and *S* the active surface area of the cantilever. On a bare silicon cantilever, a SiO_2_ surface, the gas molecule binds to the Si–OH groups through two or three hydrogen bonds. Assuming about 8 Si–OH groups/nm^2^, the estimated gas adsorption capacity is around 3 molecules/nm^2^, or 3×10^6^ molecules/μm^2^. For controlling the adsorption rate, *t_dyn_* should be faster than the time governed by thermodynamic adsorption. Basically, for an adsorption of 1 ppm of gas, *V* must be higher than 1 L/min. The flow rate can then be maintained constant at 15 mL/min during experiments. Higher flow rates could cause turbulence, or unsteady cantilever vibrations [4].

### Measurements in Liquids

In liquids, the injection is usually performed using a cell of small volume (around 1 mL) using a syringe (Figure 1 E) associated eventually with a closed loop flow. A low flow of around 1mL/min is usually used [89]. In liquids, cantilevers can also be directly integrated with conventional micro fluidic systems [7]. Stereolithography has been recently used to fabricate 3D and high aspect ratio microstructures for microfluidics. The advantage is to eliminate the dead volume of the reaction chamber and to decrease the assembly time [90]. Microsystems comprising a multiplexed array of 20 silicon microcantilevers and a polymer microfluidic system for delivery of the samples was developed for nucleic acid hybridization detection [17]. Due to the small volume of the chamber, diffusion needs to be taken into account in the adsorption kinetics [42]. Another advantage of microfluidics is the ability to accelerate the chemical reactions by a factor of 20 μL allowing the capability of handling concentrations of 100nM [91].

The cantilever response can be read out by several methods depending on the static or dynamic mode of operation but also on the spring constant of the cantilever. The cantilever deflection can be easily measured using a position sensitive detector (PSD) monitoring the reflection of a laser beam from the cantilever. The cantilever curvature can also be obtained optically with subnanometer resolution with a processing speed of about ten cantilevers per second [92].

For cantilevers with low spring constants, the dynamic response, *i.e.*, the resonance frequency *f* and the quality factor *Q* of the resonance peak, can be obtained from the vibrational noise spectrum [93] obtained with a spectrum analyzer. The effective spring constant of cantilevers may be determined using thermal noise driven resonance frequency. The amplitude and the width of the thermal noise peak are also in agreement with the integrated noise energy of the oscillator of around *k_B_ T*.

For cantilevers with higher spring constants, when the deflection is not easily detectable, the cantilever can be excited mechanically (using a piezoelectric transducer [94]), magnetically [10], or electrostatically [95]. In certain cases, due to potential reactions with aggressive gases, piezoelectric transducers used for the excitation of the cantilever must be placed outside the measuring cell. The signal from the reader is sent to a lock-in amplifier for analysis.

## 3. Elastic parameters

The elastic parameters, Young's modulus E and Poisson's ratio? *ν*? are difficult to dissociate experimentally from the reduced Young's modulus E* given by:

(2) $$E^{*}=\frac{E}{1-\nu^{2}}$$

### Static approach using single material cantilevers

In the static mode, E* can be deduced from a measurement of the cantilever's spring constant (k) by applying a force F at the end of the cantilever and measuring the corresponding deflection Δz. ko must be corrected if *F* is not exactly located at the end of the cantilever but at a distance ℓ from the end.

**Figure 2.**
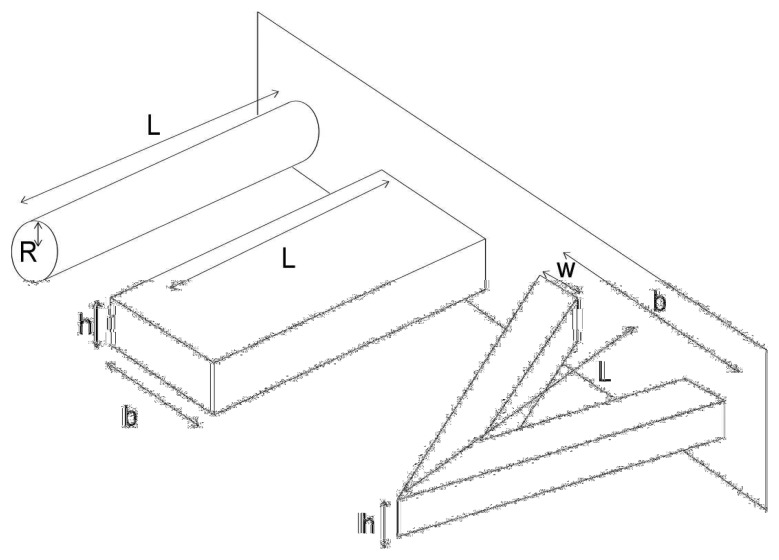
Geometrical parameters of cylindrical, rectangular and V shaped cantilever.

We therefore have:

(3) $$k=\frac{F}{\Delta z}$$,

(4) $$k=k_{o}{\left(\frac{L}{L-l}\right)}^{3}$$,

(5) $$E^{*}=\frac{1}{3}\frac{L^{3}}{I}k$$

where, *L* is the cantilever length. The shape of the cross-section of the cantilever (Figure 2) is described by the moment of inertia *I*. Denoting the cylinder radius with *R*, the cantilever width with *b*, and the cantilever thickness with *h*, we have [96]:
**Cylindrical beam****Rectangular beam****V-shaped cantilever**
(6)$$I=\frac{1}{6}R^{4}$$
(7)$$I=\frac{1}{12}bh^{3}$$
(8)$$I=\frac{1}{6}bh^{3}\left(1+4{\left(\frac{w}{b}\right)}^{3}\right)$$

The uncertainty in the evaluation of the Young modulus increases with the complexity of the system. Errors of 3-5% have been found for cylindrical cantilevers, whereas using a simplified model to describe the V-shaped cantilevers, results in 25% uncertainty [97]. The accurate metrology of the geometric parameters of cantilevers, especially with respect to thickness measurements, is known to be hard. Actually, *k* cannot be determined easily with less than 10% uncertainty for complex geometries [98]. The best approach [99, 100] is offered by a Finite Element Analysis [74, 101]. The preceding equations assume perfectly rigid support behavior and are valid in the small deformation regime. Both these assumptions can be easily violated in many microscale tests, and numerical analyses using finite-element techniques, which solves the necessary equations beyond the small deformation limit, are necessary to interpret the data.

### Static approach for a bilayer cantilever

Bilayer cantilevers, *e.g.*, those with a coating, also appear to be promising for the determination of the Young modulus of the coating [102]. The bilayer cantilever can be considered as two springs in parallel.

The resultant spring constant k_bilayer_, can then be shown to be given by the more complex expression:

(9) $$\zeta =\frac{1}{2}\frac{Eh^{2}-E_{f}{h_{f}}^{2}}{Eh+E_{f}h_{f}}$$

(10) $$k_{1}=E_{f}h_{f}b\left(\frac{{h_{f}}^{2}}{3}+h_{f}\zeta +\zeta^{2}\right)+\text{Ehb}\left(\frac{h^{2}}{3}-h\zeta +\zeta^{2}\right)$$

(11) $$k_{2}=-E_{f}{bh_{f}}^{3}\left(1+3\zeta /h_{f}+3{\left(\zeta /h_{f}\right)}^{2}+{\left(\zeta /h_{f}\right)}^{3}\right)$$

(12) $$k3=E_{f}b\left(h_{f}+\zeta \right)\left(2\zeta h_{f}+\zeta^{2}+{h_{f}}^{2}\right)$$

(13) $$k_{\text{bilayer}}=3\frac{k_{1}+k_{2}+k_{3}}{L^{3}}$$

where E_f_ and hf are the effective Young modulus and the thickness of the film, respectively.

**Figure 3.**
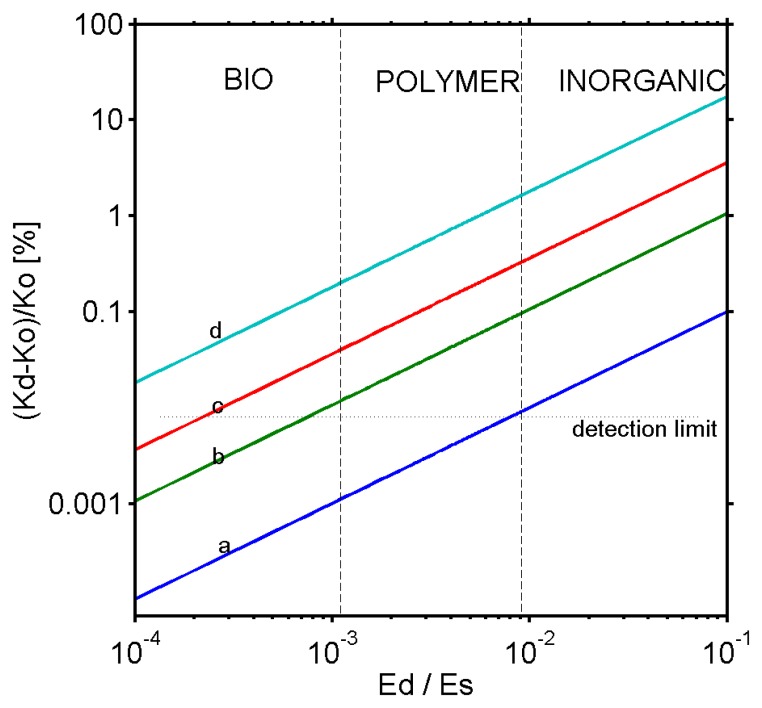
Changes in the effective spring constant of a bilayer cantilever Kd compared to a bare cantilever Ko as a function of the ratio between the Young's modulus of the coating Ed and that of the cantilever Es for various thicknesses of silicon cantilever a) h_f_/h = 3.10^-3^, b) h_f_/h = 3.10^-2^, c) h_f_/h = 1.10^-1^, d) h_f_/h = 3.10^-1^. Calculations were performed for a typical silicon cantilever having a length L = 200 μm, a width b = 40 μm and a thickness of h = 300 nm. This corresponds approximately to a spring constant of 5mN/m and a mass of 4.8 ng.

Figure 3 shows the effect of the film deposition on the spring constant of a cantilever. For a soft coating, such as used in biosensors, the spring constant of the cantilever does not undergo any measurable changes during the adsorption (less than 0.1%). On the contrary for inorganic coatings having E_f_ > 1 GPa, a subsequent change above 0.1% can be detected even for ultra thin films.

### Dynamic approach for single material cantilevers

The second method used to determine the Young modulus is the mechanical tuning method. The resonance frequencies of a micromachined cantilever has been extensively employed in the determination of the elastic modulus 103 of thin films [75].

The equation governing the cantilever motion, that is, the time dependent deflection *z* at a point *x* along the cantilever of length *L*, mass *m*, and spring constant *k*, is given by:

(14) $$\frac{kL^{3}}{3}\frac{\partial^{4}z\left(x,t\right)}{\partial x^{4}}+\frac{m}{L}\frac{\partial^{2}z\left(x,t\right)}{\partial t^{2}}=0$$

The corresponding eigenfrequencies are given by:

(15) $$\omega_{n}=K_{n}^{2}\sqrt{\frac{EI}{\rho A}}$$

where *n*=0,1,2,…

As a first approach, the problem may be approximated using a simple mechanical harmonic oscillator with the frequency:

(16) $$f=\frac{1}{2\pi }\sqrt{\frac{k}{m}}$$

From a measurement of the fundamental mode and depending on the cantilever geometry, the effective Young modulus can be determined. Both changes in the spring constant *k* and the effective mass *m* of the cantilever must be considered. A geometrical correction factor is used to account for the fact that, in the simplified model, the cantilever mass is not distributed. Equivalent mass and Young's modulus for cantilevers of most common geometries are summarized below.


Cylindrical beamRectangular beamV-shaped cantileverEffective mass*m* = 0.243PL(π*R*^2^)*m* = 0.243PL(*bh*)*m* = 0.163rL(*bh*)Effective Young's modulus
(17)$$E*=\frac{4\rho }{3}{\left(\frac{\pi L^{2}}{R}f\right)}^{2}$$
(18)$$E^{*}=0.243\rho {\left(\frac{4\pi L^{2}}{h}f\right)}^{2}$$
(19)$$E*=\frac{0.326\rho }{1+4{\left(\overset{w}{\;}/\underset{b}{\;}\right)}^{3}}{\left(\frac{2\pi L^{2}}{h}f\right)}^{2}$$

Determination of resonance frequencies of small cantilevers seems to be the most suitable way for estimating the Young modulus. This is particularly useful since, signals measured in frequency domain often display sharp peaks allowing for very small frequency shifts to be measured. But as the cantilever dynamics is very sensitive to the environmental conditions, the measurements are preferably performed in a vacuum chamber. The accuracy of the measurements may further be studied by the use of eigenmodes of higher frequencies.

The experimental procedure is based on the periodic excitation of the fixed end of the cantilever and the detection of its natural resonance frequency *f*. Measuring *f* and assuming known sample geometry and density permit therefore a determination of the mechanical properties. shows a typical frequency spectrum obtained for a rectangular cantilever under vacuum. The resonance frequencies are well described by the eigenmodes; the first mode is accurately calculated, whereas the following modes are systematically underestimated (Table 2). Note that the first eigenmodes of the cylindrical cantilevers are more accurate and the discrepancy between the results decreases with increasing number of modes *n*.

Although the vibration energy of the cantilever is less for the second mode than for the first mode, the bending angle is larger at the cantilever end and therefore more easily detectable by an optical method. hod operating in the MHz range.

Table 3 shows that cantilever measurement of the sound velocities for various metals yields values that do not exceed the well established ultrasonic values by more than 5%. The method is then reliable even if all cantilever results appear to be underestimated compared to the ultrasonic method operating in the MHz range.

### Dynamic approach using bilayer cantilevers

If we consider a bilayer rectangular beam, the effective Young modulus of the film E_f_ can be obtained from the eigenfrequencies of the uncoated (*f*) and the coated cantilever (*f_f_*).

The effective mass density of the bilayer cantilever is given by:

(20) $$\rho_{\text{eff}}=\frac{\rho h+\rho_{f}h_{f}}{h+h_{f}}$$

**Figure 5.**
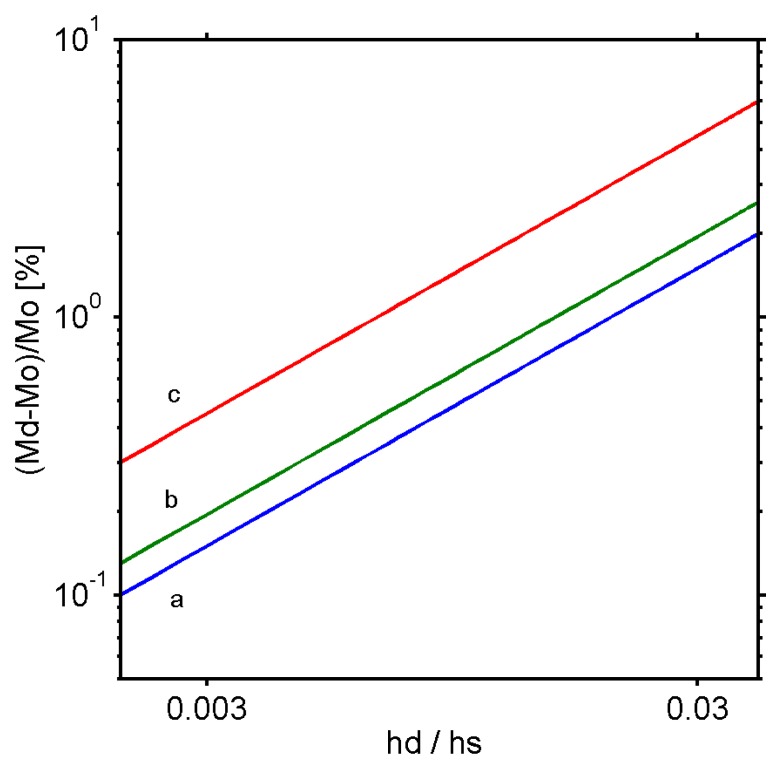
Changes in the cantilever mass M as a function of the ratio between the coating h_d_ and the cantilever h_s_ thicknesses in the case of a) bio (*P*_d_/*P*_s_ = 0.5), b) polymer (*P*_d_/*P*_s_ = 0.65), and c) metal (*P*_d_/*P*_s_ = 1.5) coating materials.

Changes in the cantilever mass as a function of the coating thickness are shown for various materials in Fig 5.

The frequencies of the coated cantilever (*f_f_*) are used to determine *E_f_*:

(21) $$E_{f}=\frac{E}{3}\left(\frac{\rho_{f}}{\rho }+\frac{2h_{f}}{h}\right)\left(\frac{1-{\nu_{f}}^{2}}{1+{\nu_{f}}^{2}-2\nu_{f}\nu }\right)\left(\frac{f_{f}-f}{f}\right)$$

As shown in Fig 5, for the bioadsorption, changes in the resonance frequency are essentially related to changes in the cantilever mass. For inorganic coatings or polymer films, the frequency is governed by both mechanical and mass changes. The mechanical properties become predominant for metals. Therefore, microcantilevers can be used to measure the Young modulus of metals. In this case, the first approximation of the cantilever as a spring (simple harmonic oscillator) can be refined when considering the residual stress.

### Poisson ratio

The Poisson ratio ν of a thin material is a very important quantity for stress analysis and structural dynamics. However, thus far, the determination of this parameter remains difficult.

The Poisson ratio is a function of the Young modulus *E* and the shear modulus *G* according to:

(22) $$\upsilon =\frac{E}{2G}-1.$$

The shear modulus *G* can be calculated from a measurement of the natural frequency of the first torsional mode *f_T_* of a rectangular cantilever using:

(23) $$G=4\frac{\rho L^{2}\left(b^{2}+h^{2}\right)}{3Dh^{2}}{f_{T}}^{2}.$$

The Poisson ratio must be considered for the flexural frequencies of relatively large cantilevers (compared to the cantilever length).

The flexural frequency is in direct proportion to the elastic parameters following the equation

(24) $$f_{r}=\frac{h}{4\pi L^{2}}\sqrt{\frac{E}{0.24\rho D}},$$

where *D* is a correction factor which depends on the width *b* to length ratio k = b/L and ν. C is expressed as:

(25) $$D=1+6.59(1+0.0752\upsilon +0.811\upsilon^{2})\kappa^{2}-0.868\kappa^{4}-\frac{8.34(1+0.202\upsilon +2.17\upsilon^{2})\kappa^{4}}{1+6.34(1+0.141\upsilon +1.54\upsilon^{2})\kappa^{4}}$$

Note that in the major cases where L ≫ b, D remains close to 1.

**Figure 6.**
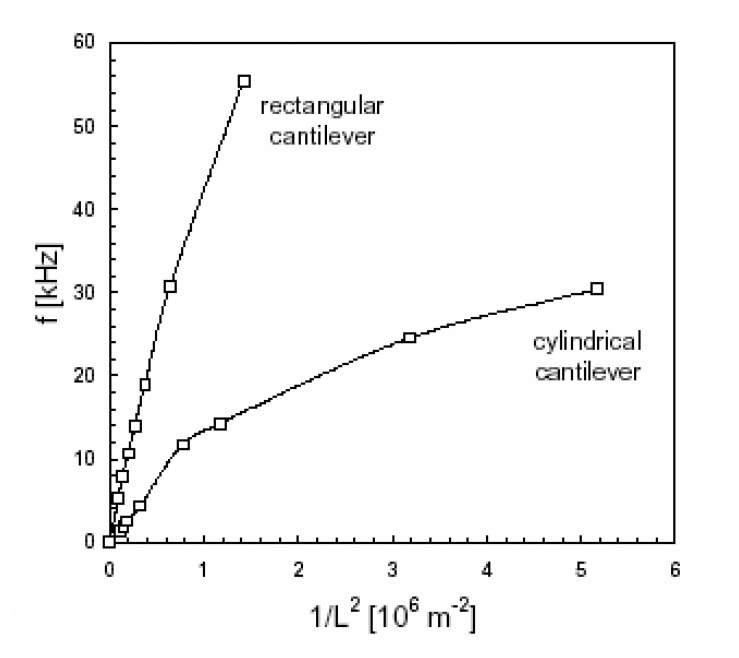
Fundamental resonance frequencies *f* of a palladium cantilever with respect to the inverse of the square length *1/L^2^* of the cantilever.

Continuum mechanics is applicable if the resonance frequency *f* normalized by *L^2^* varies linearly with the cantilever thickness *h* without a characteristic discontinuity. The *1*/*L^2^* dependence of the resonance frequency *f* (Figure 6) reveals a nonlinear behavior for shorter cantilever lengths. For *L* < *9 mm*, a correction factor *C* involved in Eq. (25) must be considered to fit all of the experimental points with a Poisson ratio of *ν*=0.3.

As the effect of geometrical parameters on the results is of great importance, only results obtained in the linear behavior in *f, i.e.*, for long cantilevers, the curve slope corresponds to the acoustic speed *(E/ρ)^1/2^*.

## 4. Inelastic parameters

This section is devoted to the measurement of the inelastic parameters of materials using cantilevers. We will first consider the effect of residual stress in simple or bimaterial cantilevers. When the cantilever becomes relatively thin compared to the cantilever length and when the cantilever coating is a monolayer, the surface stress must be considered. Anelastic behavior and fracture strength are also discussed.

### Residual stress and yield strength

Residual stress is a tension or compression, which exists in the bulk of a material without application of any external loads. The residual stress can vary from -500 MPa to 500 MPa.

The main factor that causes this stress is the grain boundary rather than the grain size. Two kinds of residual stress are therefore usually defined: the macro stress corresponds to the behavior of few grains whereas the micro stress deals with sub-microscopic areas, within a grain.

Residual stress may be created during the manufacturing process of a material, or it may accumulate in a structure over many years in operation. In either case, this stress can have a serious negative effect on a product's quality, durability and lifetime. Accurate detection of residual stress is an important element of the quality control process and helps predict the service lifetime of the product [104].

To illustrate, we note that the residual stress depends strongly on the film thickness: the highest compressive stress is created in the first 200 nm of a deposited film and the stress is relaxed significantly if the film gets thicker than 350 nm. A structure with many crystal defects can also generate a stress, which can be minimized by annealing. At high temperatures, the atoms can rearrange themselves, thus the number of crystal defects decreases, thereby reducing the stress. Generally, compressive residual stress is benefic for the fatigue life since it delays crack initiation and propagation. Tensile stress on the contrary reduces the mechanical performance of materials. Such phenomena can be at the origin of the observed asymmetric oscillations of coated cantilevers.

The residual stress can be represented by a uniform stress s _R_ and a gradient stress. The uniform residual stress is relieved though the free end of a single material cantilever. This component can be measured using a bilayer cantilever, in which the residual strains in the two materials are different leading to a bending moment. The bilayer cantilever is usually bent by several micrometers, corresponding to a radius of curvature R_s_ given by:

(26) $$\frac{1}{R_{\sigma }}=\frac{2\Delta z}{3L^{2}}$$

which can vary from micrometers up to millimetres for perfect structures. The residual stress within the film can be obtained from the original Stoney equation, derived for a beam flexed by a uniformly stressed film:

(27) $$\sigma_{f}=\frac{E_{s}h^{2}}{6h_{f}}\left(\frac{1}{R_{\sigma }}-\frac{1}{R_{o}}\right)$$

where *s*_f_ is the film's normal stress, *E*_s_ is the Young modulus of the substrate, *h* is the cantilever thickness, and *h*_f_ is the film thickness. 1/*R_s_ and* 1/*R_o_* are respectively, the curvature caused by intrinsic stress after and before the deposition. In two-dimensional film-substrate systems, the film stress *s*_f_ is deduced by replacing *E*_s_ with the biaxial modulus E_s_/(1-ν_s_), where ν_s_ is Poisson's ratio of the substrate.

Assuming σ_f_ to be uniform, the radius of curvature *R_s_* should be constant. This equation was also applied locally to calculate the film stress point-by-point, when the curvature is not constant. In these cases, the constant curvature 1/ *R_s_* is replaced by the local curvature, which depends on the positions of data points and the measured directions. Although the local application of Stoney equation is widely used, its validity cannot be established.

A more realistic approach consists in considering the uniform stress for both the bare cantilever s _R_ and the coated one s_Rf_. The cantilever will bend but it is still difficult to isolate s _R_ and s _Rf_. The radius of curvature is given by:

(28) $$R_{\sigma }=-\frac{\frac{\frac{E}{E_{f}}+{\left(\frac{h_{f}}{h}\right)}^{3}}{6\left(\frac{h_{f}}{h}+{\left(\frac{h_{f}}{h}\right)}^{2}\right)}+\frac{1+\frac{E_{f}}{E}{\left(\frac{h_{f}}{h}\right)}^{3}}{6\left(\frac{h_{f}}{h}+1\right)}+\frac{h_{f}}{2h}+\frac{1}{2}}{\sigma_{Rf}-\sigma_{R}\frac{E}{E_{f}}}Eh$$

The gradual residual stress causes the single material cantilevers to bend. The detection of the bending of single material cantilevers provides then a convenient method to measure the internal stress gradient:

(29) $$\nabla \sigma_{R}=\frac{d\sigma_{R}}{dh}=\frac{E}{1-\upsilon }\frac{2}{L^{2}}z$$

The residual stress can be also obtained from the resonance frequency. For a bridge, that is, a beam supported at both ends we have:

(30) $$\sigma_{R}=\frac{\pi^{2}Eh^{2}}{12h^{2}}\left(\frac{48\rho L^{4}}{Eh^{2}\pi^{2}}f^{2}-1\right)4\rho L^{2}f^{2}$$

**Figure 7.**
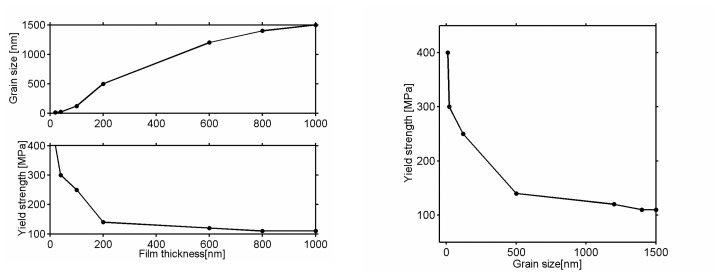
Changes in yield strength with the film thickness and film grain size.

Cantilevers have been used to determine the yield strength. The limit of validity of the elastic regime for thin films is thickness dependent, especially due to the changes in the microstructures related to the fabrication process. As the thickness is reduced, the yield strength increases with usually a decrease in the grain size. The yield strength appears to be constant for a film thicker than 1 μm (Figure 7). For small-scale structures, the elastic strain gradient should be considered. The rigidity exhibits an inverse squared dependence on the beam's thickness [105]

### Surface stress

Surface stiffness can be viewed, in a top down perspective, as a residual stress near the surface when the thickness of the film tends to zero. This stiffness can arise, for instance, from the surface roughness of the cantilever or some localized mechanical defects [21]. The cantilever can be treated as an effective mass in parallel with two springs, one linked to the bulk properties and the other one to the surface stress.

On the other hand, from a bottom up point of view, the surface stress finds its origin at the molecular scale. It [53] corresponds to the variation of interfacial energy U with respect to the strain e:

(31) $$\sigma_{s}=\frac{1}{\ell_{s}}\frac{dU}{d\varepsilon }$$

where *U* is related to the molecular potential and ℓ*_s_* the distance between the surface molecules. The surface stress of a cantilever can be deduced without knowledge of its Young modulus [52]. The measurement of surface stress can be controvertial, specially within dynamical regimes [104], where adsorption-induced changes in the spring constant may result in errors in the adsorbed mass calculated from shifts in the resonant frequency.

As previously noted, mass effects due to molecular adsorption do not contribute to a significant shift in the resonance frequency. The mass of a monolayer will result in a shift of a few Hz. However, shifts of around 100 Hz for a monolayer have been measured. Such results suggest that molecular interactions somehow affect the resonance frequency. To describe this effect, simultaneous measurements of the resonance frequency and the adsorption-induced cantilever bending have been used to determine the variation in the spring constant. Plotting the change in surface stress as a function of the chemical concentration, the surface excess of adsorbed molecules and, therefore, the mass adsorbed can be determined [55]. Change in the cantilever stiffness were first estimated for antigen-antibody interactions [106].

The surface stress can be viewed as the sum of two contributions: one is an axial force per unit length and the second is a moment (N.m) per unit cross section. A variation in the moment induces a cantilever bending but not a frequency shift. If the central part of the beam is under compression, the surface must be under tension, and the forces are balanced. No shear stress exists between the bulk and the surface layer, except at the very end.

In a first order approximation, Stoney's equation may be used to estimate the differential stress Δs from the cantilever deflection *?z* given by:

(32) $$\Delta z=\frac{3(1‐\nu )L^{2}}{h^{2}\;E}\Delta (ds)$$

where *L* and *h* are the length and the thickness of the cantilever, respectively, ν the Poisson ratio, and *E* is the Young modulus of the substrate.

Typical values of surface stress encountered are around 30 mN/m in the case of gas adsorption, 50 mN/m in the case of thiol binding, and 10 mN/m in the case of protein binding. However, inadequate modeling can lead to significant error in the estimation of the surface stress: around 10% in microscopic experiments, whereas for macroscopic cantilevers, the surface stress could be overestimated by a factor of 5 if the mass effect is neglected [107]. The original derivation of Stoney equation was then refined, in order to account for the cantilever shape [52, 57, 108, 109], and the size for MEMS [110] when the residual strain is not uniform along the thickness of the cantilever.

The effect of surface morphology on the surface stress such as the surface roughness is also controversial. Unlike prior reports that suggest the surface roughness enhances adsorption-induced stress, we observe that nanometer-size roughness may slightly decrease the adsorption kinetics and the associated surface stress [111].

The changes in the axial force can be used to determine the surface stress [112] by measurements of the resonant frequency [113]. The effect of surface stress on the resonance frequency of a cantilever sensor was modeled analytically by incorporating strain-dependent surface stress terms [114] for pure surface stress and an adsorption-induced surface stress. The effect of the pure surface stress can be considered as negligible. The equation governing the cantilever is modified to introduce the surface tension s _s_ in [108]:

(33) $$EI\frac{\partial^{4}z(x,t)}{\partial x^{4}}+\sigma_{s}\omega \frac{\partial z(x,t)}{\partial x}-\sigma_{s}\omega \left(L-x\right)\frac{\partial^{2}z(x,t)}{\partial t^{2}}=0$$

leading to:

(34) $$\sigma_{S}\approx \left[{\left(\frac{f^{\prime }}{f}\right)}^{2}-1\right]\frac{EI\pi^{2}}{12L^{3}}$$

(35) $$\frac{kL^{3}}{3}\frac{\partial^{4}z(x,t)}{\partial x^{4}}+\frac{\partial }{\partial x}\left(\sigma_{s}x\frac{\partial z(x,t)}{\partial x}\right)+\frac{m_{\text{eff}}}{L}\frac{\partial^{2}z(x,t)}{\partial t^{2}}=0$$

(36) $$\sigma_{s}\approx \frac{\pi k}{3}\frac{\Delta f}{f}$$

The changes in the surface tension are thus of the order of the spring constant for the more flexible cantilevers. A frequency shift of 50 Hz for a cantilever with k=0.5 N/m leads to a surface tension of 1 mN/m. In the dynamic method, the adsorption-induced surface stress appears to be less than that obtained by the static approach of Stoney.

### Internal friction, fracture and fatigue

When the cantilever is excited, it reaches a new mechanical state, and this equilibrium does not appear instantaneously, consistent with the observation that the corresponding relaxation time is not zero. The time lag between the response of the cantilever and the periodic excitation gives rise to a hysteresis loop. Since the hysteresis is accompanied by energy dissipation, the “compliance” or “rigidity”, relating the stress and the strain, may be defined via a generalized complex elastic modulus:

(37) $$E(\omega )E^{\prime }(\omega )+i\;E^{\prime \prime }(\omega )$$

The behavior of the cantilever is characterized by the response function E(ω), where the real part E′(ω) describes the energy stored by the cantilever and the imaginary part E″(ω) describes the energy dissipated by the cantilever.

The phase angle *F* is then given by the ratio between *E′(ω)* and *E″(ω)*. The mechanical quality (*Q*-factor) of the cantilever is defined from the internal friction denoted *Q^-1^*= *tan ϕ*. If the energy losses are low, *tan ϕ* rflects the variation of *E″(ω)*.

*Q* measured from the resonance response of a cantilever yields information on the internal friction of the material in vibration [115]. It is related to the energy absorbed per cycle of cantilever oscillation. The total losses of the system include those of the lever *Q_c_* and those of the accompanying experimental devices *Q_a_*. We note that *Q_a_* can strongly modify the apparent value of the quality factor *Q*, which may be obtained in 3 ways:

by fitting the vibration amplitude in frequency domain using the following formula:
  (38) $$\frac{Z_{c}}{Z_{p}}=\frac{1}{\sqrt{{\left(\omega_{f}^{2}-\omega^{2}\right)}^{2}+\frac{4\omega_{f}^{2}\omega^{2}}{Q^{2}}}},$$
  where *ω_f_ is the* resonance frequency, *Z_p_* vertical extension of the piezoelectric crystal and *Z_c_* the displacement of the free end of the lever;
  by the direct use of the resonance peak by measuring its width Δ*f* at the $1/\sqrt{2}$ of the maximum amplitude and by applying the relation:
    (39) $$Q=\frac{f}{\Delta f}$$
by the decrement logarithmic curve *d* defined by
  (40) $$\delta =\ln\left(\frac{{\left|Zc\right|}_{n}}{{\left|Zc\right|}_{n+1}}\right)$$,
  where *n* is the index of the *n^th^* peak after the lever has been displaced from its equilibrium position to oscillate freely while decaying exponentially back to its equilibrium position through a series of transient oscillations. Typically, *Q* can vary between 10 and 10000 depending on the nature of the cantilever.

The following equation may be considered when distinguishing *Q_A_*, the quality factor of the apparatus and *Q_c_*, the *Q*-factor of the cantilever:

(41) $$\frac{1}{Q}=\frac{\frac{1}{Q_{c}}+\frac{K_{P}}{K_{c}Q_{A}}{\left(\frac{Z_{P}}{Z_{c}}\right)}^{2}}{1+\frac{K_{P}}{K_{c}}{\left(\frac{Z_{P}}{Z_{c}}\right)}^{2}},$$

where *K_P_* is the stiffness of the piezoelectric crystal and *K_c_* is that of the cantilever, both expressed in N/m.

Cantilevers have been used to determine the dependence of the internal friction on temperature in the range between -50 to 150°C. A slight decrease in damping *Q^-1^* indicates the occurrence of a structural rearrangement of the film. Internal friction as low as 10^-5^ in micrometer thick metal films in the temperature range 300–800 K has been measured [75]. The dependence of the internal friction on the temperature, the frequency and the thickness of the film provide information on the origin of mechanical losses. The activation energy points to a dragging mechanism of jogs accompanied by vacancy diffusion along the dislocation core. This knowledge becomes important for the design of reliable MEMS devices, especially the cognition of the strength distribution of the structural material. Strength is determined by the distribution of the flaw size (around 100 nm for Si) and hence can be influenced by grain size, microstructure, and etching processes [116].

When residual stresses are sufficiently large, they can lead to fracture or delamination either after processing or during the application of sub-critical loads. For instance, fracture strength of polysilicon in uniaxial tension could vary between 2.2 and 4.3 GPa, depending upon the details of the fabrication process.

Flexural elements such as cantilevers are naturally concerned with the effects of cyclic loading on material failure [84]. Since Si does not exhibit any dislocation activity at low homologous temperatures, there is little evidence for extrinsic toughening mechanisms observed in some brittle materials. Si should not exhibit fatigue at room temperature. However, fatigue in a polysilicon device has been observed [117]. The resonance frequency of a reversed bending structure was used to monitor crack growth at a notch or precrack. Applied bending stress amplitude was plotted as a function of the cycles to failure to generate fatigue stress-life. While more than 10^8^ cycles to failure were observed at a stress amplitude estimated to be 3 GPa, this was reduced to 10^5^ cycles at a stress amplitude estimated to be 4 GPa. Environment-assisted cracking of the oxide layer is thought to cause crack growth that ultimately leads to failure. In the presence of aggressive environments, environmentally-accelerated fatigue behavior is important [116, 118].

## 5. Reliability of mechanical measurements

The reliability of the mechanical measurements will depend on both the accuracy of the measurements and the validity of the continuum mechanics. Analysis of the literature shows controversial measurements; for instance, measurements of *E* using micro cantilevers can vary over a broad range from 150 GPa up to 290 GPa [42, 59, 119-123].

The frequency *f* can be determined with an accuracy of 0.01%. The cantilever geometry is usually determined by scanning electron microscopy (SEM). Among the current experimental calibration methods for measuring *k*, none appears to be superior to the others. The thermal fluctuation method [124, 125] can be viewed as simple but the effect of interference with other noise sources is usually ignored [126]. The mass attachment [127] does not need the cantilever geometry to be known but can be destructive. Measurement of *k* from the resonance frequency [57, 128] is the simplest method which is only valid for rectangular beams, k is strongly dependent on the thin cantilever thickness. The use of another reference spring in contact with the microcantilever appears to be the most appealing method [42, 129], although delicate and time-consuming to set up. A new method based on micro drop evaporation was proposed for the determination of the spring constant [130].

The model for calculating Young's modulus presupposes an isotropic material with a constant thickness, no surface roughness, and no texture effects. These effects may cause a mean variation in the measured signals. Laser acoustic methods are insensitive to microscopic methods such as nanoindentation or microcantilevers.

A large difference in the measurement of the Young modulus for sprayed coatings was found for the cases of tension and compression, which was explained in terms of microcracks [131]. The most common error in the measurement of these properties comes from the boundary between the cantilever and the rigid support. Details of the compliance of the support should be taken into account.

It was necessary to experimentally confirm the influence of the dimensions of the cantilevers on their fundamental frequency of resonance *f_0_*. The thinner the cantilever, the more important the effects of surface and size. The limiting factors below which the macroscopic theory is no longer valid were determined. The theoretical frequencies were compared with the frequencies measured in experiments using rectangular cantilevers of thicknesses varying from 3 to 250 μm. The widths *b* and the lengths *L* were chosen so that $\frac{b}{L}<0.2$. The variations of the theoretical frequency are deferred according to the thickness *h* of the cantilever used. This variation with the value of reference is 10% for *h* = 3 μm and decreases with increasing thickness except for a key thickness (20 μm in this case) that can be correlated with the microstructure of the material: the average size of the grain is 500 nm; the bigger grains, of a size of 1.5 μm, account for approximately 5% of the total quantity of grains. The instantaneous frequency deviations according to the thickness of the cantilevers and the material microstructure converge towards the same conclusion.

To apply the mechanics of the continuous media require that the smallest dimension of the sample, in fact the thickness of the cantilevers, is at least 20-fold larger than the larger characteristic dimension of material, that is to say the grain size for a polycrystalline solid. Variation of the reference resonance frequency remained lower than 2% as long as *h*>20 μm, a value corresponding to a ratio of approximately 20 between the thickness and the largest characteristic dimension, given by the grain size. Measuring the bulk mechanical properties correctly will imply, thereafter, to choose a cantilever thicker than 20 μm.

The nonlinear response of a cantilever at large deflections is sometimes also overlooked. A general study of cantilever beam nonlinearity under a variety of loading conditions was performed with analytical and finite element analyses. The cantilever nonlinearity was found to increase with increasing cantilever deflection. The linear analysis was found to underestimate the applied load by up to 15% [132].

## 6. Measurement of the environmental properties of cantilevers

Variations in the environmental parameters of the cantilever, namely the change in temperature and the pressure surrounding the cantilever can strongly affect the cantilever response compared to its behavior in high ultra vacuum.

### Temperature

The double-layer microcantilevers are very sensitive to the variations in temperature because of the “bimetallic” effect in connection with the difference between the thermal dilation coefficients of various materials. The cantilever deflection becomes then extremely sensitive to temperature changes. Two temperature modes were distinguished:

For small temperature changes Δ*T* (< 3°C), the deflection Δz varies linearly with Δ*T* in agreement with the thermal coefficients of gold *a_Au_* or/and the constituent silicon nitride *a_S_* of the microcantilever such that:

(42) $$\begin{array}{l} \Delta z=3\left(\alpha_{Au}-\alpha_{s}\right)\left(\frac{c+1}{B}\right)\left(\frac{L^{2}}{t_{s}}\right)\Delta T\\ \begin{array}{ccc} c=\frac{t_{Au}}{t_{s}}, & B=4+6c+4c^{2}+\varphi c^{3}+\frac{1}{\varphi c}, & \varphi =\frac{E_{Au}}{E_{s}} \end{array} \end{array}$$

where *t_s_* and *t_au_* are the thicknesses of silicon nitride and the gold layer, respectively.

For higher temperatures, the sensitivity in temperature is attenuated due to non linearity of the cantilever response. In addition to the sensitivity in deflection, the sensitivity *S_T_* in frequency of resonance [48] has two origins: first, the sensitivity of the Young modulus of each material with the temperature, and second, the bimetallic effect stretching the layers out, and we have

(43) $$\begin{array}{cc} S_{T}=\frac{df}{f_{0}dT}=\frac{\alpha }{2}+\frac{dE}{2\text{EdT}}\;, & \alpha =\frac{\alpha_{Au}\alpha_{s}}{\alpha_{Au}+\alpha_{s}} \end{array}.$$

The temperature sensitivity is thus at the origin of new types of sensors [4] by coupling electrical and thermal measurements: cantilever-type integration of a piezoresistor device for simultaneous sensing of the bending, ramping the temperature, and controlling the temperature cycles. With a mass resolution in the picogram range, this approach can outperform current thermogravimetric methods by five orders of magnitude. Due to its small size, thermal time constants as low as 1 μs can be reached and adjusted via the cantilever geometry and material properties [133].

Microcantilevers with quantum wells were also fabricated for manipulating, in real-time, the energy states, thus providing photon wavelength tunability. Applications were then found in an effective and rapid change in electron energy levels for photon detection devices, such as InSb microcantilevers and small arrays of GaAs/GaAlAs microcantilever. Uncooled Infrared (IR) radiation detector were then designed at room temperature [134, 108, 135, 93]. Local thermal analysis was then achieved using heated silicon atomic force microscopy probes for a thin film of polystyrene [136] and using tapered optical fibers [137].

### Pressure sensitivity, Knudsen's number, Reynold's number

The pressure effect on microcantilever is clearly visible in the dynamic mode but also in the static mode for both gas and liquid environments.

### In gases

**Figure 9.**
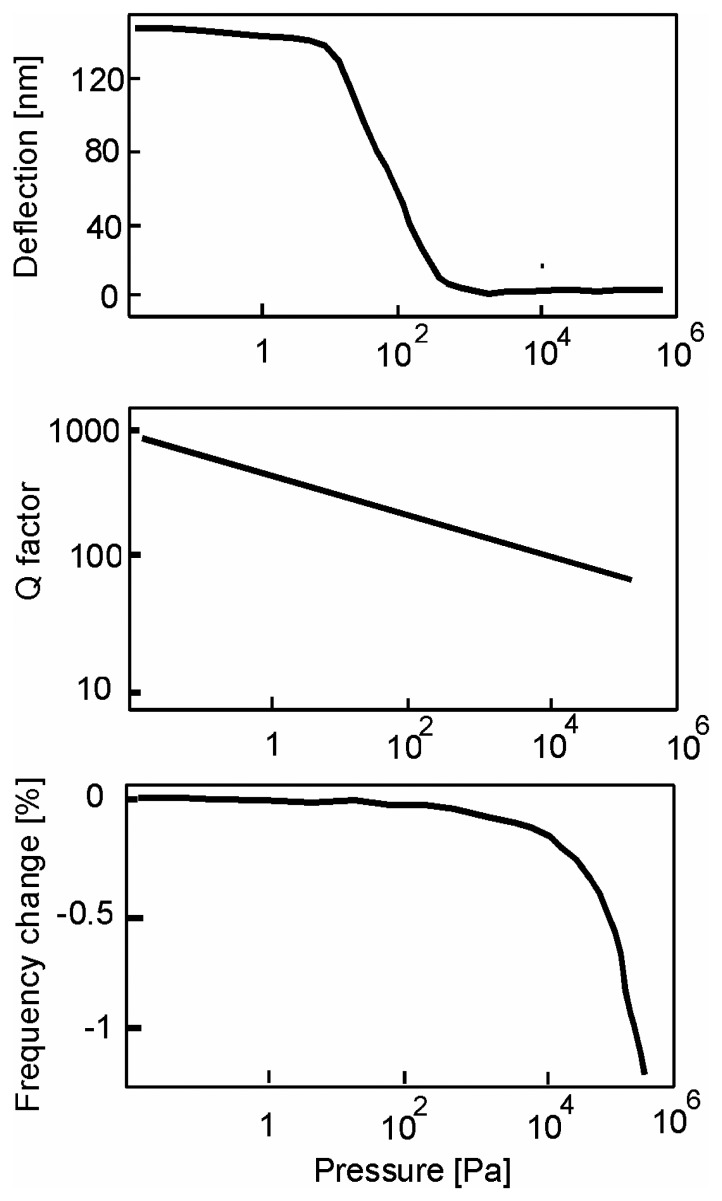
The pressure dependence of the deflection, the *Q*-factor, and the resonance frequency of the cantilever [2]

Various gases, such as helium and nitrogen with pressures between 10^-2^ and 10^5^ Pa were used to investigate specific molecular properties (Figure 9).

Among the various flow characteristics, the Knudsen number *K_n_* is the most significant:

(44) $$\kappa_{n}=\frac{\lambda }{b}=\frac{1}{d_{g}\sigma_{c}b},$$

Where λ is the mean free path of the gas molecules, b the cantilever width, dg the density of the gas (air=1)?σ_c_ the cross section of collision of the molecules.

Three regimes are distinguished: the molecular regime (*K_n_*>10), the transitional regime (10 > *K*_n_ >0.01), and the viscous regime (*K_n_*<0.01).

In the molecular mode, the properties of the gas, considered as rarefied, are difficult to reach by macroscopic parameters like the temperature. If no variation in frequency and temperature is detected, the *Q*-factor decreases according to:

(45) $$\begin{array}{l} \Delta Q=A_{1}\sqrt{\frac{R_{g}T}{fM\eta P}}\\ f=\sqrt{{f^{2}}_{0}-\gamma^{2}}=f_{o}\sqrt{1-\frac{1}{2Q^{2}}} \end{array}$$

where A_1_ is a geometrical constant, *R_g_* is the constant of perfect gases, M is the molar mass of the gas and η its viscosity, P is the pressure, and *f_o_* is the resonance frequency under ultra high vacuum.

In the viscous regime, the intermolecular collisions control mainly the gas properties. The cantilever acceleration is the paramount parameter determining the frequency dependence of the resonance; the increase in the pressure induces the uptake of effective mass of the cantilever. In this mode, the frequency of resonance and the quality factor vary in accordance with:

(46) $$\begin{array}{l} f=f_{0}\sqrt{\frac{1}{1+\frac{A_{2}MP}{\rho R_{g}T}}}\\ \Delta Q=A_{3}\sqrt{\frac{\text{fRT}}{\eta MP}} \end{array}$$

Q decreases in an identical way in the viscous and molecular regimes. The deflection being stable in this regime, the temperature can be considered as constant in this zone.

The transitional regime, suitable for the passage from the molecular mode to the viscous mode, is explained by the equilibrium between the effects of the speed and the inertia. *Q* is then disturbed, but this zone is especially well described by the signal of deflection. The deflection is thus explained not by an adsorption of helium but primarily by heating effects. In the molecular mode, the cantilever temperature is not necessarily identical to that of the gas molecules at the surface; thermal balance is reached only with the transitional arrangement with a sufficiently strong density of gas molecules. The nitrogen having a thermal conductivity lower than helium causes less cantilever bending confirming the heating effects of the transitional arrangement.

### In liquids [38, 138]

In liquids, the cantilever motion is damped by a viscous term. The damping can be used to determine the viscosity?? and the density ? of very small volumes of fluids. The Reynold number is used to account for the geometry of the vibrating cantilever as well as in the description of the viscous properties of the liquid.

The general equation of motion of the cantilever in a medium can be written as:

(47) $$EI\frac{\partial^{4}z}{\partial x^{4}}+\left(D+D_{F}\right)\frac{\partial z}{\partial t}+\left(M_{1}+M_{2}\right)\frac{\partial^{2}z}{\partial t^{2}}=0,$$

where *E* is the Young modulus, *I* is the moment of inertia of the cantilever, *x* the coordinate along the cantilever, *D* is the intrinsic damping of the cantilever (internal loss) which can be determined separately under vacuum. *D_F_* is the fluid damping coefficient describing the energy loss in the fluid. *M_1_* is the mass per unit length of the cantilever, *M_.2_* the added mass due to the fluid (mass of fluid moving along with the cantilever per unit length), and *z* is the cantilever displacement at distance *x* from the fixed end of the cantilever.

The damping due to the viscosity of the fluid is given by:

(48) $$D_{F}=\frac{b}{b}\left(1+\frac{4}{\sqrt{2{\text{R}}_{\text{e}}}}\right),$$

where R_e_ is the Reynold number of the fluid, which depends on the angular frequency ω and is defined as:

(49) $$R_{e}=b^{2}\omega \;\left(\overset{\rho }{\;}/\underset{\eta }{\;}\right).$$

The solution of the equation of motion is a complex quantity. The angular resonance frequency is given by:

(50) $$\omega =\sqrt{\frac{3.52^{2}EI}{(M_{1}+M_{2})L^{4}}}+i\;\left(\frac{D+D_{F}}{2(M_{1}+M_{2})}\right)=\omega_{r}+i\omega_{i}\;,$$

where ω*_r_* and ω*_i_* can be deduced from the measurements of the resonance frequency f and the quality factor Q under liquid according to:

(51) $$\begin{array}{ll} {\omega_{r}}^{2}=\frac{f^{2}}{1-Q^{2}} & {\omega_{i}}^{2}=\frac{Q^{2}f^{2}}{1-Q^{2}}. \end{array}$$

Therefore, *M_2_* and *C_V_* can now be written as:

(52) $$M_{2}=\frac{3.52^{2}EI}{{\omega_{r}}^{2}L^{4}}-M_{1},$$

(53) $$D_{F}=2(M_{1}+M_{2})\omega_{i}-D,$$

Initially the cantilever can be calibrated in vacuum or in a fluid with known properties for determining its intrinsic resonance properties. Later the cantilever can be resonated in unknown fluids.

## 7. Applications

Cantilevers provide the opportunity to develop a new method for the identification of material damage and/or for the experimental verification appropriate for the evolution of the damage laws [139]. We review here our recent applications in metallurgy such as palladium tritide materials, in buildings materials such as cement, in composite materials such optical fibers, or organic materials. Determination of the ageing process using cantilevers is found to be particularly relevant.

### Palladium tritide cantilevers

Palladium cantilevers were used to investigate the issue of the storage of tritium, a radioactive isotope of hydrogen [77]. A good means to store tritium is to form a metal tritiure, PdT_0.6_ palladium, thereby ensuring a valuable storage in terms of compactness (7 liters TPN of hydrogen in 9 cm3 of palladium) and safety (low equilibrium pressure, approximately 50 mbar at room temperature). The performance of this type of device with time, i.e., the ageing process of metal tritiures, requires the determination of the evolution of their physicochemical properties.

### Isotopic effect (Hydrogen, Deuterium, Tritium) on Young's modulus

**Figure 10.**
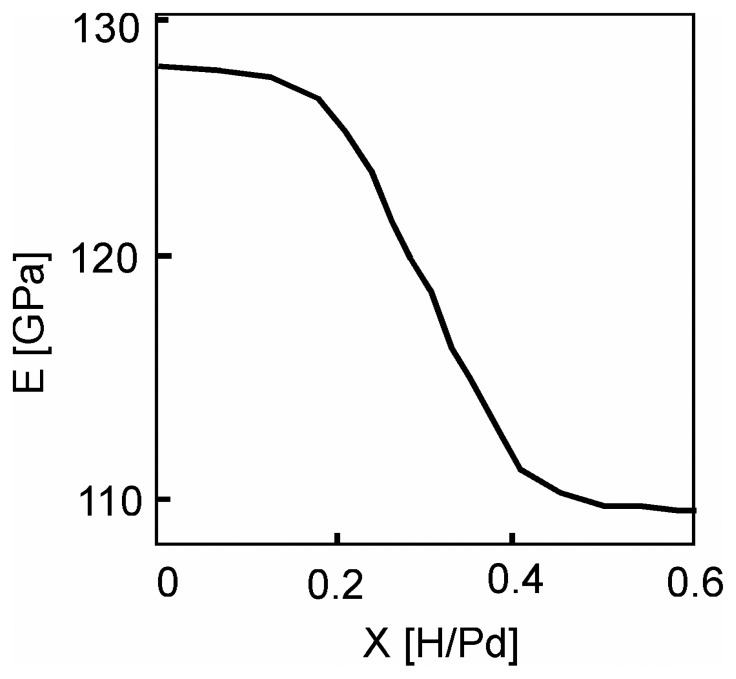
Changes in the Young modulus of palladium cantilever as a function of the stoichiometry *x* =*H/Pd*.

Young's modulus *E* of the palladium was measured using vibrating cantilevers according to the pressure of hydrogen, deuterium and tritium. In situ measurements enable one to monitor the changes in *E* of the hydride phase as a function of the stoichiometry *x*=*H*/*Pd* (Figure 10).

When the palladium is completely hydrided in β phase (PdH_0.6_), a 10% swelling in material volume occurs; *E_x_* for the hydride is then obtained from:

(54) $$E_{x}={\left(\frac{2\pi {L_{x}}^{2}f_{x}}{r_{x}}\right)}^{2}\frac{\rho_{x}}{3}=\frac{4\pi^{2}}{3}{\left(\frac{\left(1+x\overset{\Delta a}{\;}/\underset{a}{\;}\right){L_{0}}^{2}f_{x}}{r_{0}}\right)}^{2}\rho_{x},$$

Where:

(55) $$\rho_{x}=\left(\frac{1+\overset{m_{H}}{\;}/\underset{m_{pd}}{\;}x}{1+3\overset{\Delta a}{\;}/\underset{a^{x}}{\;}}\right)\;\rho_{0},$$

with *f_0_* and *f_x_* being the resonance frequencies of the Pd and its hydride, respectively. *L_0_*, *r_0_* and *L_x_*, *r_x_* correspond to the length and radius of the cantilevers Pd and its phase hydride, $\overset{\Delta a}{\;}/\underset{a}{\;}=5.33.10^{-2}$, m_H_ = 1.008 g.mol^-1^ and m_Pd_ = 106.42 g.mol^-1^.

Cantilevers with rectangular and circular cross sections were hydrogenated. The circular cross section was preferred for dynamic analysis since rectangular sections induce irreversible bending by gradients of residual stress generated by the hydrogen insertion differing from one cantilever face to the other.

**Figure 11.**
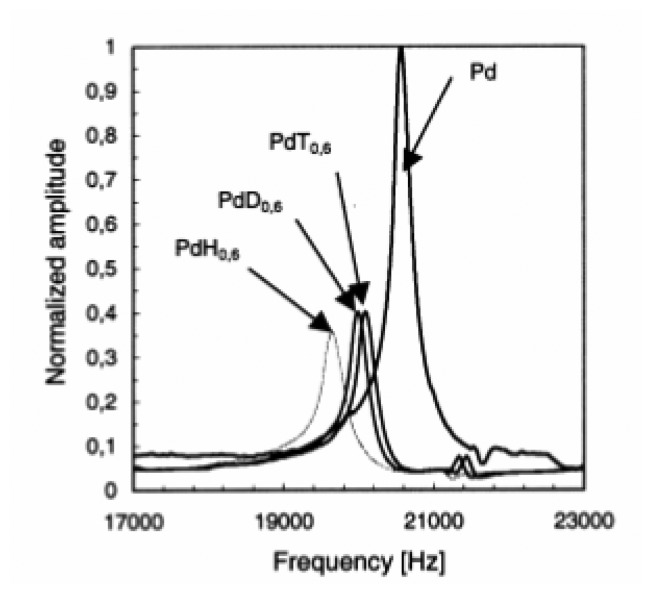
Resonance frequency peaks of a cylindrical palladium cantilever under vacuum, hydrogen, deuterium and tritium.

An isotopic effect (Figure 11) on the properties of the material was thus established. The change in the Young modulus (*E_PdH_*> *E_PdD_*> *E_PdT_*) was explained in terms of optical phonons, strongly related to the isotope mass.

Let us consider the radioactive decay of PdT_x_, helium-3 atoms are produced following the equation $\frac{1}{2}T_{2}\to^{3}He^{+}+\beta^{-}+\overset{-}{\upsilon }$, where? *β* is an electron and υ̅ an electronic anti-neutrino. Every 12 years, half of the tritium atoms present in the octahedral sites of palladium is transformed into ^3^He [140]. This relatively short time duration is at the origin of a considerable quantity of ^3^He (1.5% at the end of three months of ageing). Being very insoluble in Pd; ^3^He tends to precipitate forming ^3^He nanobulles with a number and a diameter growing with time. The elastic properties of PdT_x_ were monitored with time under a tritium pressure. The ageing process of several microcantilevers enables one to demonstrate that during the first days, the Young modulus of PdT_0.6_ increases approximately by 2% before stabilizing after one month [141].

### Ageing process of optical fibers

One of the limitations in the communications by optical fibers is the mechanical resistance in the long run of fibers in aggressive and varied environments such as underwater or underground spaces in the subway. The ageing of optical fibers with respect to moisture or temperature is not completely understood. Hydrogen H_2_ at the origin of the growth of defects (Si-O-Si + H_2_0 → Si-O-H, Si-O-H) seems to induce the most dramatic degradations. Several mechanical models based on the finite elements, discretizing the fiber by elements of 1 mm length, were developed to analyze the problems of fracture. The resonance frequency was used to determine the unknown Young modulus of these fiber elements [142].

The influence of the radius of the fiber core was studied using 2 monomode fibers (*r_core_* = 3.5 and 10 μm) and a multimode fiber (*r_core_* = 50 μm). The composition laws for the elastic modules of the composites (Voigt model) were studied to obtain:

(56) $$E_{\text{composite}}=\left(\frac{1}{V_{\text{core}}+V_{\text{clading}}}\right)\left(E_{\text{core}}V_{\text{core}}+E_{\text{clading}}V_{\text{clading}}\right),$$

as a function of the volume V of the clading and the core.

**Figure 12.**
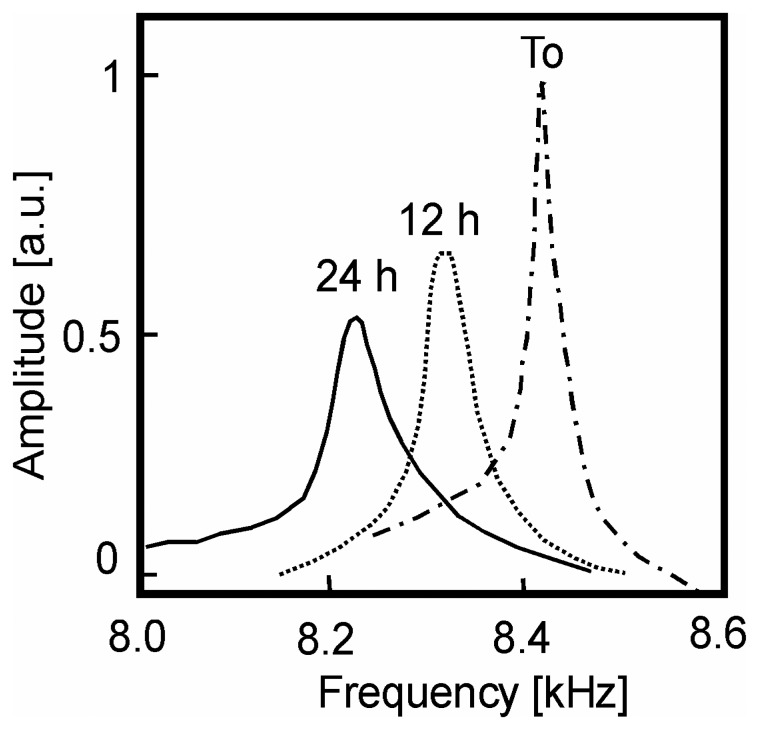
Temporal evolution of the frequency spectrum of a monomode optical fiber under a hydrogen bar.

The effective Young modulus *E_composite_* of various fibers were then deduced from the fundamental frequencies of vibration and compared with different volumes. The reactivity of optical fibers with hydrogen was monitored in time showing a linear drop in frequency (30 Hz per minute) during the first 20 hours, with a gradual reduction in the *Q*-factor (Figure 12). An AFM study does not reveal any surface modification after the hydrogen exposure, confirming that the phenomenon takes place in the bulk of the fiber. The frequency drop is not connected to the mass uptake of the hydrogen but to the degradation in the mechanical properties: 4% frequency shift corresponds to 2% loss in Young modulus *E*.

### Strengthening of cement cantilevers

The mechanical setting [143-145] of the cement pastes is known to be very slow, the miniaturization of the samples fortunately helps accelerate the process. The mortar is interesting since this composite material is made up of a porous cement matrix and rigid inclusions such as sand. Its specificity stems from the interface between the grains and the matrix; a discontinuity responsible for the mechanical properties of the mortars [146].

The determination of the role played by such interfaces remain however difficult by conventional methods (rheology, techniques involving an inflection of the beam, or ultrasonic) requiring samples of centimeter size. Since hydration is highly exothermic, the miniaturization of the samples on a scale lower than the millimeter is recommended, especially for better modeling of the inclusion/matrix interface. Mortar based cantilevers have been fabricated with millimeter lengths and micrometric thicknesses.

Resonance frequencies of the mortar cantilevers cover an interesting range between 1 and 100 kHz corresponding to a region of the spectrum that has not been explored by either rheology (Hz) or ultrasonics (MHz). We therefore sought to investigate the various contributions to the elastic properties of the composite cantilevers to determine the influence of the parameters such as the hydration time, the porosity of the cement paste and the inclusion concentration within the matrix. The formalism of the continuous media could still be applied to the pure cement cantilevers thicker than 1 μm. For mortar cantilevers, namely including glass balls of 40 μm in diameter, the minimal thickness was fixed at 300 μm.

**Figure 13.**
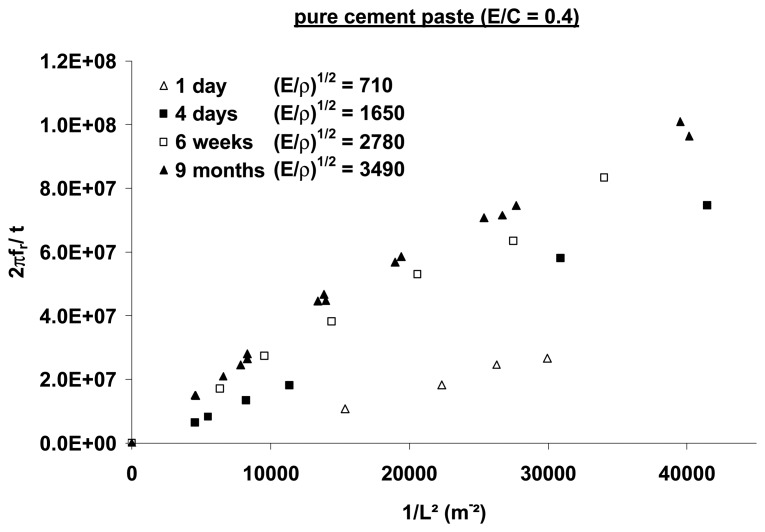
Evolution of the resonance frequency f of the cement cantilever standardized by the thickness h according to the reverse of the square length *L* of the cantilever for various setting times. The pure cement paste was characterized at the origin by a water content of 40%.

The viscoelastic limit was determined by requiring that the deflection of 5 mm long cantilevers must not exceed 75 μm to satisfy the deformation criterion of 0.02%. Results were analyzed in terms of acoustic speed, measured very precisely starting from the variations of the resonance frequency *f* standardized by the thickness *h* according to the inverse of the length square, i.e., *1*/*L^2^*. The absolute determination of the effective Young modulus *E* of the material remained approximate (about 25 GPa) accounting for the approximate knowledge of the density (2 kg/m^3^) of the miniaturized levers. The evolution of the resonance frequency with the hydration time (Figure 13) shows that prior to 4 days, the mechanical properties strongly evolve due to the percolation in the cement paste; then during the ensuing 9 months, the increase in *E* weakens because of the filling of the pores by the hydrates reinforcing the structure. Material porosity was modified via the volumetric ratio between the water and the cement.

### Measurement of changes in surface tension and film stress

#### Irreversible surface tension induced by chemical etching

Variations in surface stress can be generated reversibly by water adsorption [112] or by etching on one face of the cantilever.

**Figure 14.**
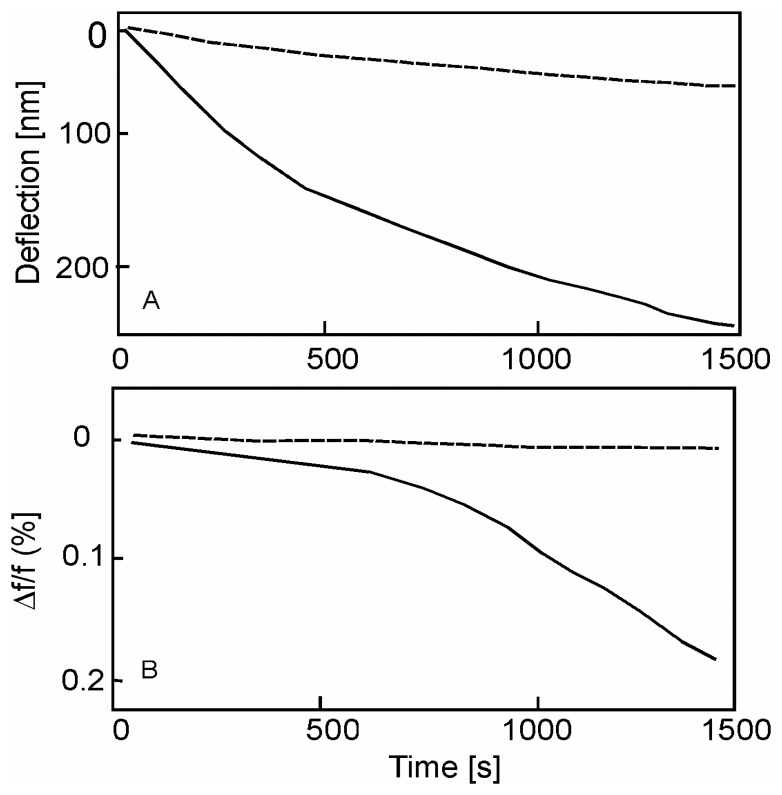
Response to HF exposure of cantilevers covered with Si_3_N_4_ films (dashed line) and SiO_2_ films (solid line) (A) in deflection and (B) in resonance frequency.

Silica is known in micro-electronics to be very sensitive to hydrofluoric (HF) acid. Microcantilevers can be used then as alternative sensors for heavy post-analysis methods. Both the deflection and the resonance frequency of the microcantilevers were analyzed according to the acid flow and concentration as shown in Figure 14 [147].

The stoichiometry and the roughness of the sensitive layers play a paramount role in the surface reactivity. For the lowest concentrations (< 10 ppm), the cantilever deflection provides the most sensitive signal. In the case of Si_3_N_4_ coatings, a linear and small variation is induced in the surface tension compared to the case with SiO_2_ coatings. Frequency shift was explained in terms of mass loss at high concentrations. The non-linearity of the deflection observed for the SiO_2_ levers arises from the etching, which initially commences on the sides, and continues in the transverse direction.

#### Film stress or swelling induced by chemical absorption

Absorption of organic vapors such as benzene and hexane in thin film sensors can lead to changes in the film stress. PECVD membranes deposited on microcantilevers are advantageous on many points: they provide continuous films without porosity, are chemically inert, and possess physically stable defects. In addition to a strong capacity for gas absorption, their high selectivity makes them very competitive in the field of polymeric membranes. For example, the selectivity of butane/methane is 4 for polymers PDMS (PolyDiMethylSiloxane) and 15 for plasma polymers. A 1 μm thick polymeric membrane (a-SiOC:H) [148, 149] was plasma deposited on a quartz microbalance (QCM) and a microcantilever for comparison.

**Figure 15.**
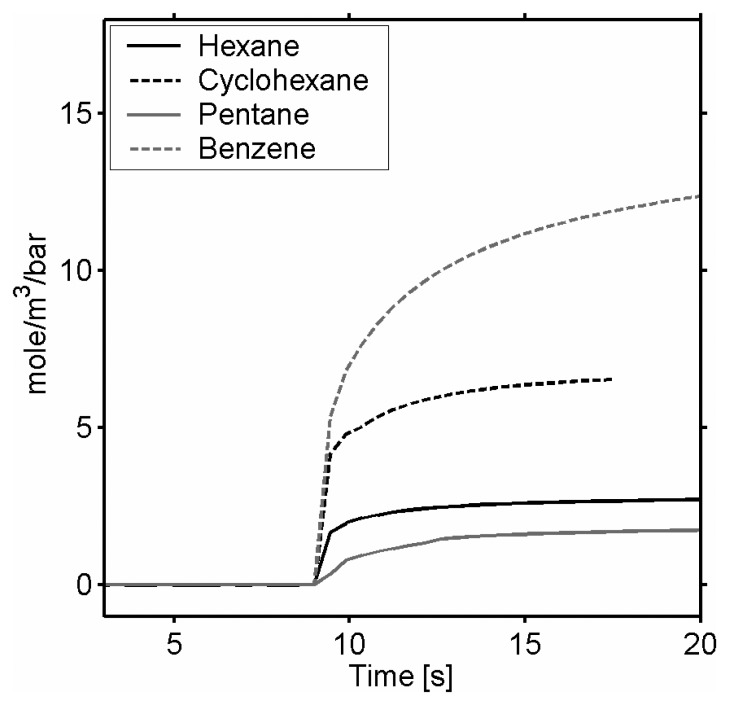
Temporal variation measured by QCM of the number of moles of vapor absorbed by the a-SiOC:H film standardized by the saturated vapor pressure for each gas and the volume of film.

The QCM (Figure 15) shows that cyclic molecules (cyclohexane and benzene), having the lowest saturation pressure, are more soluble in the film than the linear molecules (pentane and hexane). Desorption is also quasi instantaneous for the linear molecules whereas cyclic molecules diffuse more slowly.

The cantilever bending was used to measure the stress variation *? σ_f_of* a polymer film deposited on the cantilever surface as a function of various gases:

(57) $$\Delta \sigma_{f}=\frac{E_{s}{e_{s}}^{3}}{3h_{f}L^{2}\left(h+h_{f}\right)\left(1-\nu_{s}\right)}\Delta z,$$

**Figure 16.**
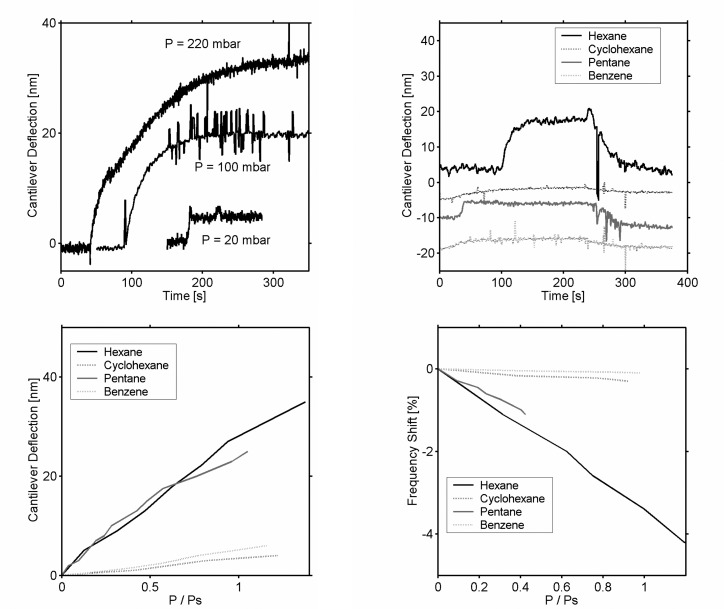
A) Absorption kinetics of hexane vapor at various pressures monitored by the bending of the cantilever coated with a-SiOC :H polymer. B) Cantilever selectivity in deflection of the a-SiOC :H polymer to hydrocarbon vapors (P=220 mbar).C) Cantilever deflection as a function of the vapor pressure standardized by the saturated vapor pressure for each gas P_S_. D) Frequency response of the cantilever as a function of the vapor pressure standardized by the saturated vapor pressure for each gas P_S_.

The tension *?s* at the interface between the film and the cantilever is given by :

(58) $$\Delta z=\frac{3L}{h+h_{f}}\left[\frac{1+\frac{h}{h_{f}}}{3{\left(1+h+h_{f}\right)}^{2}+\left(1\frac{hE_{s}}{h_{f}E_{f}}\right)\left(\frac{h^{2}}{{h_{f}}^{2}}+\frac{h_{f}E_{f}}{hE_{s}}\right)}\right]\frac{\Delta s}{\left(\frac{E_{s}E_{f}}{E_{s}+E_{f}}\right)},$$

where *L* is the cantilever length, *h* and *h_f_* are the thicknesses of the lever and the polymeric film, and *E_s_* and *E_f_* are the Young moduli of the cantilever and the polymer. A deflection *Δz* of 10 nm corresponds to a variation of tension of 0.75 N/m. The deflection of the lever varies linearly with the gas pressure with a sensitivity of 2 nm/10 mbar. The fast response, for small variations in the pressure, can last 10 min for larger pressures (200 mbar) (Figure 16 A).

Concerning the selectivity of the polymeric films, the hexane induces clearly the most important bending (Figure 16 B). Cyclohexane is not so easily detectable contrary to the QCM measurements; complementarities of the techniques thus become obvious. The origin of the tension of film becomes understandable when plotting the cantilever deflection as a function of the gas pressure standardized by the vapor pressure of each gas (Figure 16 C). The most influent parameter is *Δσ* the variations of film swelling and the elastic properties explain the slight variations in the linearity of *Δz*/*ΔP*. All the linear molecules seem to induce identical variations in tension, but especially more important than the cyclic molecules, certainly more compact, they may condense between the aggregates. The increase in deflection means that the film tension decreases; the condensation of gases between the grains increasing the intergranular distance can be at the origin of the softening of film.

The study reproduced in the dynamic mode confirms the selectivity with the vapors already noticed in static mode (Figure 16 D). Interpreting the major reduction in frequency (4%) just in terms of mass uptake seems delicate. The QCM measurements indicate a 20% mass uptake of polymeric film, namely changes in cantilever mass of 0.3%. The mass effect contributes only to 10% of the frequency shift. Variations in the film tension appear to be the major contribution in *Δf*/*f*. The spring constant of the cantilever *k* having been measured to 36 N/m, was also initially obtained from estimating the deflection *Δσ* to 2.6 N/m; the variations *Δk*/*2k* = 3.6% agree with the observed frequency shift. The resonance frequency reflects consequently the mechanical properties of the polymer layer.

## Conclusions

In summary, we have shown that cantilevers provide genuine tools for the investigation of the mechanical properties of small volumes of materials and their temporal evolution under gaseous environments.

As temperature gauges, double-layered microcantilevers operating in deflection mode, can reach extreme thermal sensitivities.

As pressure sensors, using properties such as the quality factor, microcantilevers operating under dynamical resonance mode, can detect various molecular modes defined by the Knudsen number..

As mass sensors, cantilevers exhibit sensitivities on the order of pg/Hz. Converted to the frequency shift per unit area; this sensitivity is 10 times higher than that generally obtained by other types of piezoelectric sensors (quartz microbalances, surface acoustic waves).

It can be concluded that even if the mass change must be considered, the high sensitivity of microcantilevers to molecular adsorption comes from the change in the mechanical properties. A rigorous analysis as a function of the size and the dynamic and the static behavior of the cantilever enables one to discern between:

The bulk properties: such as the change in the Young modulus, which can be measured by analyzing the resonance frequency response. This was illustrated by the studies of the ageing process of three materials: metal tritides, optical fibers, and composite materials such as cement.

Film and interface properties such as the residual stress, and the surface stress were studied by the etching or the absorption process using polymeric or inorganic thin films.

Future trends will consider smaller cantilevers to investigate the mechanical properties of nanosized cantilevers in the MHz regime. Silicon and carbon cantilevers are promising for further exploration. The use of other materials having electro or photomechanical abilities will bring the subject one step further. Simultaneous analysis of the mechanical response of the cantilevers with local electrical and/or optical properties is also an interesting challenge.

## Figures and Tables

**Figure 4.**
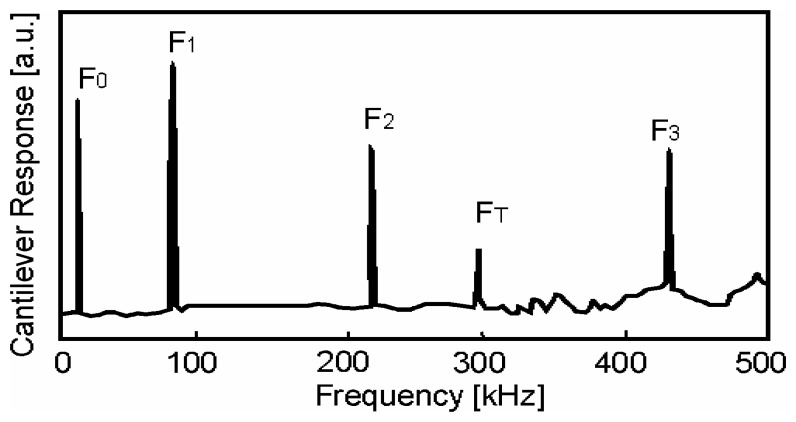
Frequency spectrum of a rectangular beam under vacuum

**Figure 8.**
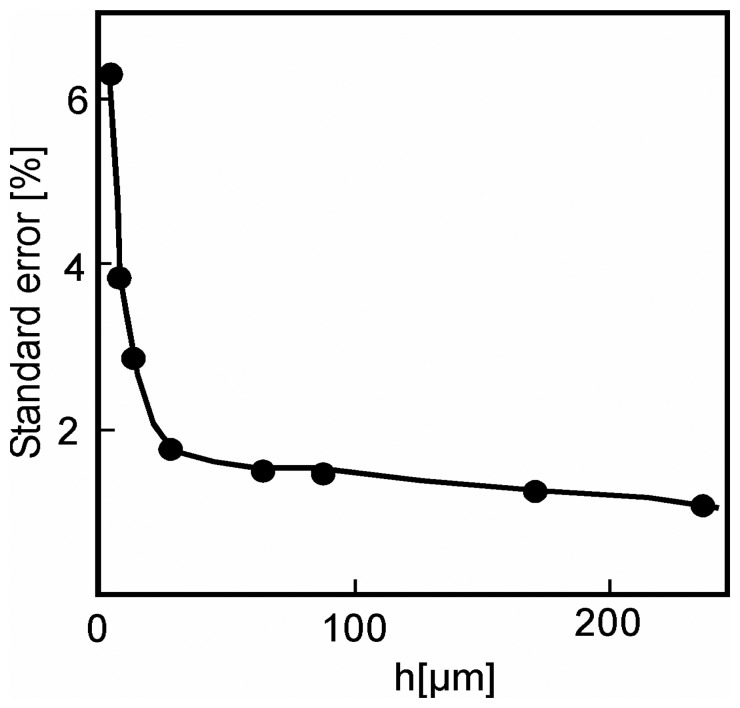
Deviation of the measured frequency from the theoretical reference frequency as a function of the thickness of the cantilever.

**Table 1.** Some mechanical parameters of soft and hard materials.

	E [84]	ν	s _Y_ (MPa)	s _F_(MPa)
LB film	anisotropic 0.3 - 2.5	anisotropic 0.1- 0.8	15-35	
Rubber	0.01 - 0.1	0.5	4-12	25
Polystyrene	2	0.35	30	30
Aluminum	70	0.33	50	710-1000
Silicon	150	0.17	300	700
Single Carbon nanotube	1000	0.17	not reached	not reached

**Table 2.** Ratio of the n^th^ frequency over the fundamental frequency. Lines 2 and 3 correspond to rectangular cantilevers and lines 4 and 5 to cylindrical ones.

**n**	**1**	**2**	**3**	**4**

b/L=0.2	6.85	19.1	36.4	62.4
b/L=0.1	6.44	18	35.4	57
d/L=0.03	6.05	16.9		
d/L=0.03	6.19	17.3		
Theory	6.27	17.6	34.4	56.9

**Table 3.** Sound velocities of some metals.

$\sqrt{\frac{E}{\rho }}(m/s)$	**Au**	**Cu**	**Pd**	**Fe**

Cantilever	6.85	19.1	36.4	62.4
Ultrasonic	6.44	18	35.4	57

**Table 4.** Frequency shift of a microcantilever with a Young's modulus *E*, mass density *P* and thickness h due to the mass uptake *Δm* for various deposited film (*E_f_*, *ρ_f_*) of varying thickness *h_f_*

***(Δm/m)/(Δf/f)* (%)**	**Biopolymer** ***E_f_*= 0.1 GPa,** *ρ_f_***= 1000 kg/m^3^**	**Inorganic/polymer** ***E_f_*= 10 GPa,** *ρ_f_***= 1500 kg/m^3^**	**Metal** ***E_f_*= 100 GPa,** *ρ_f_***= 2000 kg/m^3^**
*h_f_/h* = 3.10^-3^	99.4%	71.4%	24.8%
*h_f_/h* = 3.10^-2^	99.3%	70,2%	23.9%
*h_f_/h* = 3.10^-1^	98.9%	58.5%	19,5%
